# Working-Memory-Guided Attention Competes with Exogenous Attention but Not with Endogenous Attention

**DOI:** 10.3390/bs13050426

**Published:** 2023-05-18

**Authors:** Ping Zhu, Qingqing Yang, Luo Chen, Chenxiao Guan, Jifan Zhou, Mowei Shen, Hui Chen

**Affiliations:** 1 Department of Psychology and Behavioral Sciences, Zhejiang University, Hangzhou 310030, China; 2 Department of Psychology, New York University, New York, NY 10003, USA

**Keywords:** working-memory-guided attention, exogenous attention, endogenous attention

## Abstract

Recent research has extensively investigated working memory (WM)-guided attention, which is the phenomenon of attention being directed towards information in the external environment that matches the content stored in WM. While prior studies have focused on the potential influencing factors of WM-guided attention, little is known about the nature of it. This attention system exhibits characteristics of two classical distinct attention systems: exogenous attention and endogenous attention, as it can operate automatically like exogenous attention yet persist for a long time and be modulated by cognitive resources like endogenous attention. Thus, the current study aimed to explore the mechanism of WM-guided attention by testing whether it competed with exogenous attention, endogenous attention, or both. Two experiments were conducted within a classic WM-guided attention paradigm. Experiment 1 included an exogenous cue and revealed an interaction between WM-guided attention and exogenous attention. Experiment 2 replaced the exogenous cue with an endogenous cue and demonstrated that endogenous attention had no impact on WM-guided attention. These findings indicate that WM-guided attention shares mechanisms with exogenous attention to some extent while operating in parallel with endogenous attention.

## 1. Introduction

Attention and working memory (WM) are two closely related cognitive systems. It has been widely investigated how attention affects WM; for example, how attention exerts substantial influences on multiple stages of WM processing [1,2,3]. Recently, more studies have concentrated on how WM affects attention, and they found that when the information in the external environment was consistent with that stored in WM, attention would be directed to it. Such attentional orienting is called WM-guided attention.

In the lab, one of the classic paradigms of studying WM-guided attention is embedding a visual search task within a WM task [4,5,6,7]. For instance, in the study of Soto and Humphreys [6], the participants were required to memorize a colored shape and complete a change detection task at the end of the trial. During the memory retention period, the participants were asked to complete a visual search by searching for a target titled line among three distractor vertical lines, each embedded within a different colored shape. While searching, one of the colored shapes in the display that never contained the target line might match the memorized item. The result showed that the search time was significantly slower in the match condition, relative to a neutral baseline where there was no match between the memory and search displays. Such a phenomenon is known as the WM-driven attentional bias effect, and it suggested that attention was captured by the information that matched what was held in WM.

WM-guided attention works in a robust manner. Many behavioral studies claimed that attention could be driven by WM, where distracting information matched to that held in WM slowed the reaction time to find the search target [4,5,6,7,8]. More directly, ERP (event-related potential) studies have demonstrated it by showing that the information matched to that held in WM could trigger the N2pc component [9,10], which is a typical electrophysiological indicator of the deployment of attention [11,12,13]. Additionally, WM-guided attention can be in operation across various memorized stimuli. Many previous studies adopted a colored shape as the memory item and found that not only the color but also the shape could guide attention [4,5,6,7]. In addition to these simple features, some other complex, meaningful information, such as faces, scenes, and real-world objects, could also guide attention [8,14]. The studies mentioned above all recruited healthy people as participants, while actually, WM-guided attention can also play a role in people with schizophrenia [15,16].

Though WM-guided attention persists in various circumstances, it is modulated by some factors. The amount of information in WM and how it is organized affect the orientation of attention [17,18,19,20]. Although many studies have consistently shown that one single representation (e.g., one color) stored in WM could produce the WM-driven attentional bias effect [4,5,6,7,19,20,21,22,23], whether multiple representations (e.g., two different colors from separate objects) stored in WM could simultaneously guide attention still remains unresolved, as they could in certain cases but not in others (guide attention [18,24,25,26]; not guide attention [27,28]). More interestingly, when multiple representations are combined into one object (e.g., two or three colors coming from one integrated object), each of them could trigger the WM-driven attentional bias effect, which is comparable in strength to the effect triggered by a single representation (e.g., one color) stored in WM [17]. Additionally, the representational fidelity of the information in WM also determines whether and how strongly it guides attention, with only highly well-represented information guiding attention automatically [29]. Although research on WM-guided attention has been conducted extensively over the past two decades, little is known about the nature of the orienting of attention driven by WM, and the mechanism underlying WM-guided attention remains unclear.

Orienting of attention can be influenced by external stimuli and internal goals, leading to the development of two distinct attention systems: exogenous attention and endogenous attention [30,31,32]. Exogenous attention, also known as bottom-up attention, is driven by the properties of the stimulus [33]. It is usually studied in the spatial cueing paradigm, wherein an exogenous cue (e.g., a flashing dot) indicating where a target will appear directs attention to that location, resulting in faster and better detection of the target [34]. Exogenous attention is characterized to be rapid and transient, with its effect typically peaking at around 120 ms after cue onset [30,35,36]. Moreover, this effect occurs involuntarily, regardless of whether the cue is below the threshold of consciousness [37] or the cue validity is at a random level [35]. Another system, known as endogenous attention or top-down attention, is directed by internal goals and expectations [34]. It is also studied in the spatial cueing paradigm, where the endogenous cue is typically a symbol such as an arrow or a number that indicates the target location [33]. Endogenous attention is characterized to be sustained, with its effect taking around 300 ms from cue onset to be effective, and it can persist as long as needed [30,36,38]. Moreover, endogenous attention operates as a voluntary system and is flexibly allocated based on cue validity [35], while its effect is subject to cognitive resource constraints [39]. In addition to the behavioral evidence mentioned above, neurophysiological evidence also supports the distinction between exogenous attention and endogenous attention. Although the brain regions activated in both attention systems are partially overlapped, exogenous attention involves the ventral frontoparietal network, while endogenous attention involves the dorsal frontoparietal network and relies on delayed feedback from the frontal to the parietal cortex [40,41,42]. In general, exogenous attention and endogenous attention are independent of each other and possess distinct natures.

WM-guided attention shares similarities with both exogenous attention and endogenous attention. On one hand, WM-guided attention can operate automatically like exogenous attention. Attention can be directed to the distracting information that matches the contents of WM, even if it disrupts the search for the target [4,5,23]. On the other hand, like endogenous attention, WM-guided attention can persist for a long time [5] and is modulated by cognitive resources. When cognitive resources are depleted, the WM-driven attentional bias effect weakens or even disappears [43,44]. Based on these similar characteristics discussed above, we hypothesized that WM-guided attention might share mechanisms with exogenous attention and endogenous attention to some extent. To test this hypothesis, we investigated the extent to which WM-guided attention interacted with exogenous attention and endogenous attention. If WM-guided attention shared mechanisms with exogenous attention and endogenous attention to some point, the WM-driven attentional bias effect should interact with the attentional effect triggered by the exogenous cue and the endogenous cue. This logic was borrowed from a study that elucidated the mechanisms of exogenous attention and endogenous attention [45], as well as from a study that assessed the nature of attention shifts in gaze-cueing [46]. Otherwise, no such interaction effect should be observed. In Experiment 1, we included the exogenous cue within the classic paradigm studying WM-guided attention. The exogenous cue was presented before the visual search task and indicated the possible location of the search target. Experiment 2 was identical to Experiment 1, except that we replaced the exogenous cue with the endogenous cue. The results showed that the WM-driven attentional bias effect was modulated by the validity of the exogenous cue rather than the endogenous cue, indicating that WM-guided attention competed with exogenous attention but not with endogenous attention and suggesting that WM-guided attention shared the mechanism with exogenous attention at least to some extent while operating in parallel with endogenous attention.

## 2. Experiment 1

Experiment 1 investigated whether WM-guided attention competed with exogenous attention by testing whether the WM-driven attentional bias effect could be modulated when the exogenous cue was valid or invalid for the search target.

### 2.1. Method

#### 2.1.1. Participants

We used G*Power 3.1 [47] to estimate the appropriate number of participants. According to a comparable previous study [22], the effect size (dz) to detect a WM-driven attentional bias effect was estimated as 0.51. The power calculation yielded an estimated minimum of 33 participants to detect the attentional capture effect with 80% power (with α set to 0.05). Thus, we set the sample size to 34 and maintained it constant across the experiments.

A total of 34 native Chinese students (10 males and 24 females; mean age = 19.1 years, SD = 1.3 years) were recruited from Zhejiang University and compensated with CNY 30 per hour or course credits. All of the participants had normal or corrected-to-normal vision and had no color vision defects. This study was approved by the institutional review board at the Department of Psychology and Behavioral Sciences, Zhejiang University.

#### 2.1.2. Apparatus and Stimuli

The stimuli were presented on a 17-inch CRT monitor with screen dimensions of 1024 × 768 pixels at a 100 Hz refresh rate. The participants were seated in a dark room and viewed the screen from approximately 60 cm away. They responded via a computer keyboard. The experiment was programmed and presented using MATLAB with the Psychophysics Toolbox extensions [48,49].

All stimuli were displayed on a gray background (RGB: 150, 150, 150). A black fixation (1.03° × 1.03°) was always presented at the center of the screen except for the memory display and the memory test display. Both the memory display and the memory test display contained one central colored square (1.15° × 1.15°), whose color was randomly selected from five colors: red (255, 0, 0), yellow (255, 255, 0), blue (0, 0, 255), green (0, 128, 0), and pink (255, 192, 203). The search display contained three distractor lines (black vertical lines, 0.43° × 0.09°) and one target line (a black tilted line, 12° either to the left or the right with equal probability). Four lines were embedded within four distinct colored hollow squares (1.15° × 1.15°) that were evenly distributed over an invisible central circle with a radius of 5.50°. There were two possible configurations of the four search stimuli locations (angle relative to the horizon: 30°/120°/210°/300° or 60°/150°/240°/330°) with an equal probability. The exogenous cue was a hollow black square (1.15° × 1.15°) that could be presented upon the location of one of the four search stimuli.

#### 2.1.3. Procedures and Design

**Figure 1:**
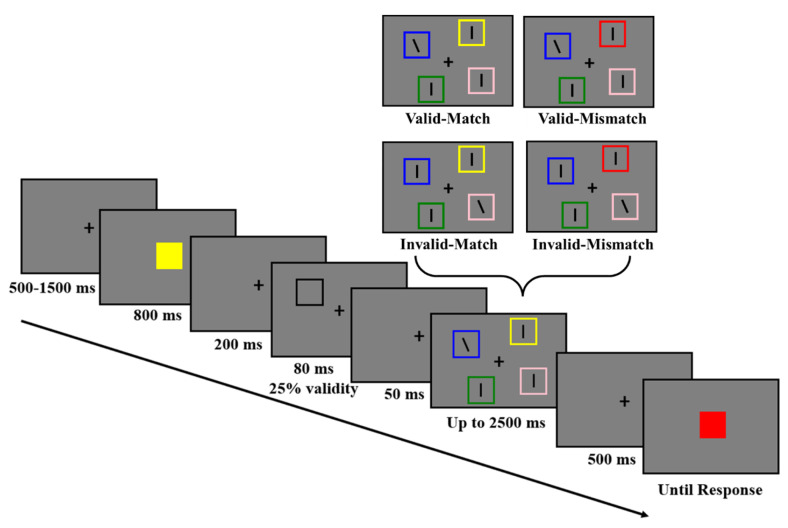
The task procedure of experiment 1. The participants were instructed to memorize the color of the memory item and complete a color change-detection task at the end of the trial. During the memory retention period, an exogenous cue was presented with 25% validity and then, the participants were required to indicate the direction of the tilted line. One of the distractors could share the same color with the memory item (match), or not (mismatch).

In Experiment 1 (see Figure 1), each trial began with a 500~1500 ms fixation, which was followed by an 800 ms memory display. The participants were instructed to remember the color of the square. After a 200 ms fixation, the exogenous cue appeared. As previous studies have shown, the effect associated with exogenous attention has a quick onset but is short-lived, and its transient peak usually occurs around 120 ms from the cue onset [30,35,36]. Thus, the exogenous cue was set to flash for 80 ms, which was followed by a 50 ms fixation. Then, the search display appeared. The exogenous cue was uninformative about the location of the search target, so the validity of the exogenous cue was 25%. Specifically, in 25% of the trials, the search target and the exogenous cue were presented at the same location (i.e., the Valid condition), while in 75% of the trials, the search target was presented at a different location to the exogenous cue (i.e., the Invalid condition) and was invalid. Additionally, there were two WM-Search Match conditions for the search distractors. In half of the trials, one of the three distractors shared the same color with the memory item (i.e., the Match condition), while in the other half, none of the search stimuli shared the same color with the memory item (i.e., the Mismatch condition). The participants were asked to find the tilted target line as quickly and accurately as possible and indicate whether the line was tilted to the left or right by pressing the left or right arrow key on the keyboard. The participants had to give a response within 2500 ms, and the search display disappeared once a response was given. After a 500 ms fixation, a memory test display was presented. The color of the test square remained the same as the memory item in half of the trials and changed to a new color in the other half. The participants were instructed to judge whether its color was the same as the memorized one by pressing “J” (same) or “K” (different) with no time limit.

Experiment 1 used a 2 (exogenous cue validity) × 2 (WM-Search Match condition) within-subject design. The participants completed at least 32 practice trials before completing the formal experiment. There were 3 formal experimental blocks of 64 trials each: 24 invalid-match trials, 24 invalid-mismatch trials, 8 valid-match trials, and 8 valid-mismatch trials, resulting in a total of 192 trials.

#### 2.1.4. Data Analysis

The primary dependent variable in this experiment was the reaction time (RT) in the search task. The data analysis procedure was aligned with previous studies that used a similar paradigm [18,22,28].

A three-step data-trimming process was applied. First, trials with incorrect responses in either the memory or the search tasks were removed. Additionally, trials with extreme search RTs (outside the range of 300–2000 ms) were also removed. Finally, trials with search RTs exceeding 3 standard deviations from the mean search RT within each condition for each participant were removed. This procedure removed 4.0% of the trials. No participant was excluded as a whole because of bad performance.

For the statistical analysis, *t*-tests (two-tailed) and repeated-measures analysis of variance (ANOVA) were performed. Additionally, Bayesian analyses were conducted using JASP [50], and Bayes factors were computed for all results. It was considered that BF_01_ provided at least moderate evidence for the null hypothesis if it exceeded 3, anecdotal evidence if it stood between 0.33 and 3, and at least moderate evidence supporting the alternative hypothesis if it was smaller than 0.33 [51,52].

### 2.2. Results

The accuracy of the search task and the memory task is summarized in Supplementary Table S1.

**Figure 2:**
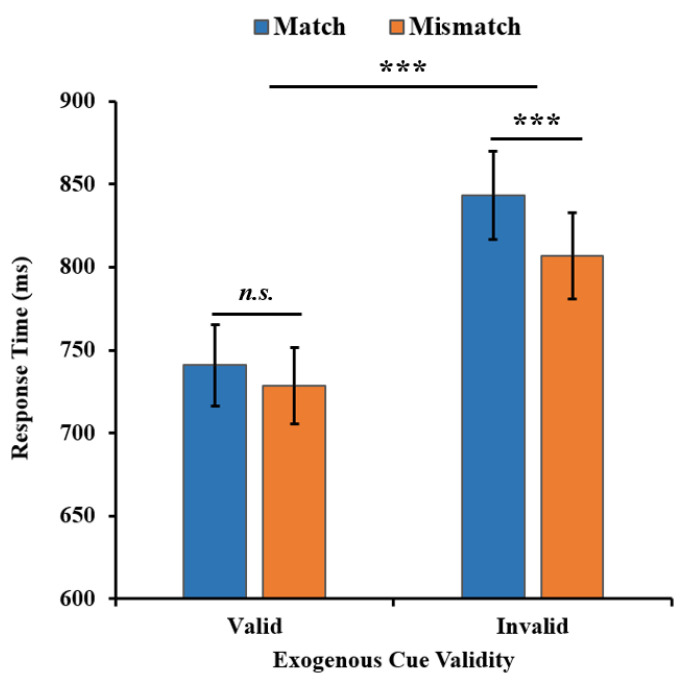
The search RT results of two WM-Search Match conditions in two exogenous cue validity conditions in Experiment 1. Error bars represent the standard error of the mean (SEM). *** *p* < 0.001. *n.s.* = not significant.

For the search RT of the search task (see Figure 2), a two-way repeated-measures ANOVA yielded a significant main effect of the exogenous cue validity (*F*(1, 33) = 87.633, *p* < 0.001, η_p_^2^ = 0.726, BF_01_ = 7.295 × 10^−9^), with a faster search RT in the valid condition than that in the invalid condition (736 ms vs. 825 ms). There was a significant main effect of the WM-Search Match condition (*F*(1, 33) = 13.389, *p* = 0.001, η_p_^2^ = 0.289, BF_01_ = 0.034), where the search RT in the match condition was slower than that in the mismatch condition (817 ms vs. 788 ms). Additionally, there was significant interaction between the exogenous cue validity and the WM-Search Match condition (*F*(1, 33) = 6.957, *p* = 0.013, η_p_^2^ = 0.174, BF_01_ = 0.291). Thus, a separate analysis of the valid condition and the invalid condition was adopted.

In the valid condition, the search RT in the match condition was not significantly different from that in the mismatch condition (mean difference [MD] = 12 ms, 95% CI [−7, 31], *t*(33) = 1.322, *p* = 0.195, Cohen’s *d* = 0.089, BF_01_ = 2.453), while in the invalid condition, the search time in the match condition was significantly slower than in the mismatch condition (MD = 36 ms, 95% CI [23, 50], *t*(33) = 5.561, *p* < 0.001, Cohen’s *d* = 0.237, BF_01_ = 1.845 × 10^−4^). The results showed that the memorized color could not produce the WM-driven attentional bias effect when the exogenous attention focused on the search target. However, there was a significant WM-driven attentional bias effect when the exogenous attention did not focus on the search target. This indicated that such an effect was modulated by the validity of the exogenous cue, suggesting that the WM-guided attention competed with exogenous attention and shared the mechanism with exogenous attention at least to some extent.

## 3. Experiment 2

Experiment 2 investigated whether WM-guided attention competed with endogenous attention. Similarly, we tested whether the WM-driven attentional bias effect could be modulated when the endogenous cue was valid or invalid for the search target.

### 3.1. Method

#### 3.1.1. Participants

The paradigm of Experiment 2 is similar to Experiment 1. thus, we decided to maintain a consistent number of participants as in Experiment 1. A new group of 34 native Chinese students (13 males and 21 females; mean age = 20.7 years, SD = 2.3 years) were recruited from Zhejiang University and compensated with CNY 30 per hour or course credits. All of the participants had normal or corrected-to-normal vision and had no color vision defects.

#### 3.1.2. Procedures and Design

**Figure 3:**
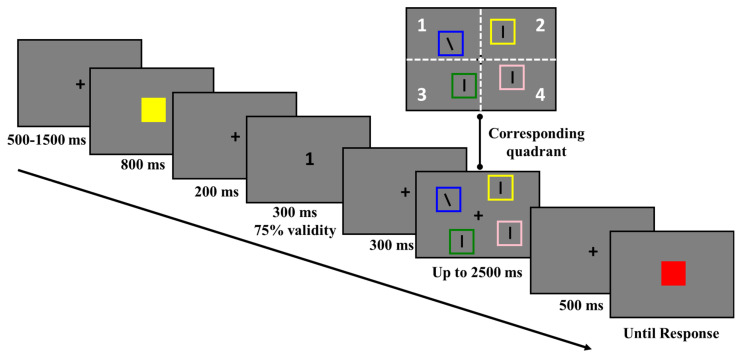
The task procedure of experiment 2. The participants were instructed to memorize the color of the memory item and complete a color-change-detection task at the end of the trial. During the memory retention period, an endogenous cue was presented with 75% validity, and the numbers 1 to 4 corresponded to the left top, right top, left bottom, and right bottom quadrant, respectively. Then, participants were required to indicate the direction of the tilted line. One of the distractors could share the same color with the memory item (match) or not (mismatch).

Experiment 2 was identical to Experiment 1, except for the following changes. In the cue display (see Figure 3), the endogenous cue was a black Arabic number (1.10° × 1.10°) presented at the center of the screen, which was chosen from 1, 2, 3, and 4 with equal possibility. The validity of the endogenous cue was 75%, and participants could use it to predict the location of the search target, with 1 to 4 corresponding to the left top, right top, left bottom, and right bottom quadrant, respectively. According to previous studies, endogenous attention usually takes around 300 ms to deploy and can be sustained over time, which allows the participants to voluntarily focus on a cued location [30,36,38]. Thus, the endogenous cue was set to present for 300 ms, and the duration of the fixation between the endogenous cue and the search task was set to 300 ms.

In Experiment 2, it was a 2 (endogenous cue validity) × 2 (WM-Search Match condition) within-subject design. The participants completed at least 32 practice trials before the formal experiment. There were 3 formal experimental blocks of 64 trials each: 24 valid-match trials, 24 valid-mismatch trials, 8 invalid-match trials, and 8 invalid-mismatch trials, resulting in a total of 192 trials.

### 3.2. Results

The accuracy of the search task and the memory task is also summarized in Supplementary Table S1.

**Figure 4:**
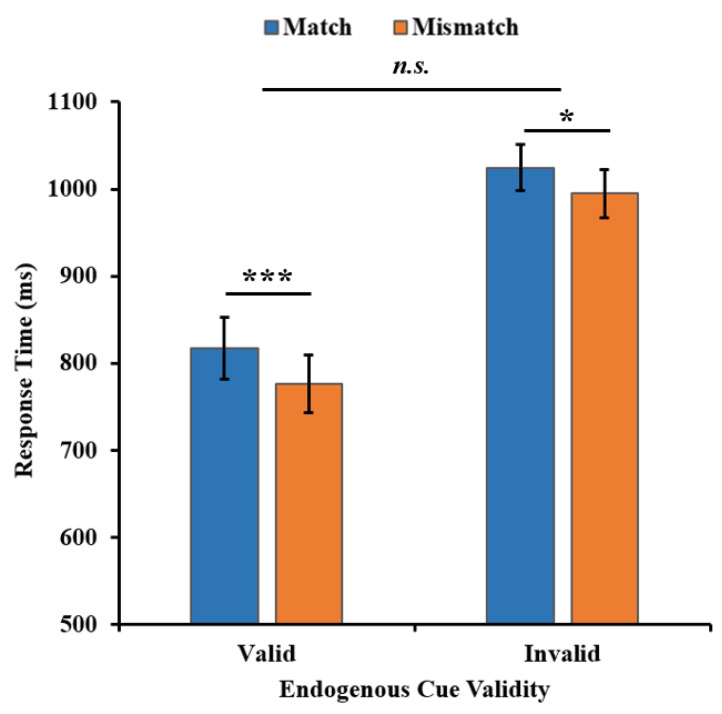
The search RT results of two WM-Search Match conditions in two endogenous cue validity conditions in Experiment 2. Error bars represent SEM. * *p* < 0.05, *** *p* < 0.001. *n.s.* = not significant.

The search RT analysis was the same as Experiment 1, resulting in 5.3% of trials being removed, and no participant was excluded as a whole because of bad performance. For the search RT of the search task (see Figure 4); a two-way repeated-measures ANOVA yielded a significant main effect of the endogenous cue validity (*F*(1, 33) = 51.402, *p* < 0.001, η_p_^2^ = 0.609, BF_01_ = 5.315 × 10^−6^), with a faster search RT in the valid condition than that in the invalid condition (794 ms vs. 1008 ms). There was also a significant main effect of the WM-Search Match condition (*F*(1, 33) = 23.328, *p* < 0.001, η_p_^2^ = 0.414, BF_01_ = 0.002), with a slower search RT in the match condition than that in the mismatch condition (865 ms vs. 828 ms). Importantly, there was no significant interaction between cue validity and the WM-Search Match condition (*F*(1, 33) = 0.917, *p* = 0.345, η_p_^2^ = 0.027, BF_01_ = 2.663). Actually, the search RT was slower in the match condition than that in the mismatch condition no matter whether the endogenous cue was valid or invalid (valid: MD = 42 ms, 95% CI [27, 56], *t*(33) = 5.732, *p* < 0.001, Cohen’s *d* = 0.206, BF_01_ = 1.157 × 10^−4^; invalid: MD = 30 ms, 95% CI [7, 53]; *t*(33) = 5.732, *p* = 0.013; Cohen’s *d* = 0.192, BF_01_ = 0.295).

The results showed that the memorized color could produce a WM-driven attentional bias effect regardless of whether the endogenous attention focused on the search target or not. This indicated that such an effect was not modulated by the validity of the endogenous cue, suggesting that WM-guided attention did not compete with endogenous attention and was in parallel with endogenous attention.

**Figure 5:**
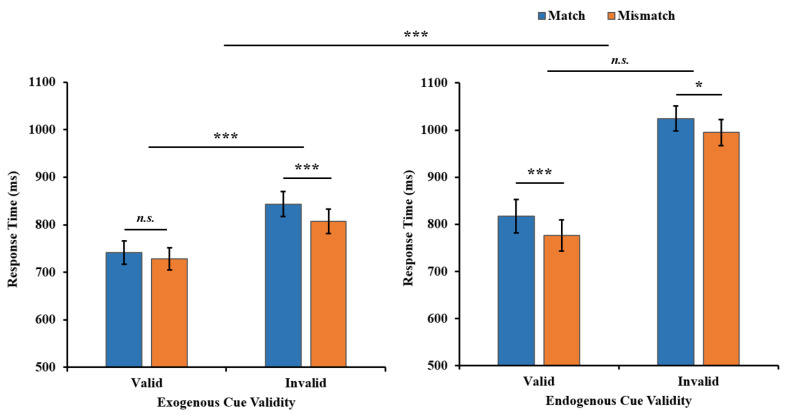
The search RT results of each condition in Experiments 1 and 2. Error bars represent SEM. * *p* < 0.05, *** *p* < 0.001. *n.s*. = not significant.

Furthermore, a between-experiment comparison was executed (see Figure 5). Taking cue validity (valid/invalid) and the WM-Search Match condition (match/mismatch) as the within-subject independent variables and cue type (exogenous/endogenous) as a between-subject independent variable, a three-way repeated-measures ANOVA yielded significant three-way interaction between the cue type, cue validity, and WM-Search Match condition (*F*(1, 66) = 5.508, *p* = 0.022, η_p_^2^ = 0.077, BF_01_ = 0.331). This indicated that the modulation of the WM-driven attentional bias effect by the exogenous cue was significantly greater than the endogenous cue, suggesting that at least to some extent, the mechanism of WM-guided attention was shared with exogenous attention but not with endogenous attention. Additional results from the three-way repeated-measures ANOVA are detailed in the Supplementary Materials.

## 4. Discussion

With two experiments, the present study sought to explore the mechanism of WM-guided attention by investigating whether WM-guided attention competed with exogenous attention and endogenous attention. Experiment 1 found that the WM-driven attentional bias effect was modulated by the validity of the exogenous cue, while Experiment 2 found that such an effect was not modulated by the validity of the endogenous cue. More importantly, the between-experiment comparison showed that the modulation of the WM-driven attentional bias effect by the exogenous cue validity was significantly greater than that by the endogenous cue validity. These results indicated that WM-guided attention competed with exogenous attention but not with endogenous attention, suggesting that WM-guided attention shared the mechanism with exogenous attention at least to some degree but operated in parallel with endogenous attention.

The results of Experiment 1 revealed a fascinating interaction between WM-guided attention and exogenous attention. Specifically, a significant WM-driven attentional bias effect was only evident when the exogenous cue was invalid, while it disappeared when the exogenous cue was valid. These results implied that WM-guided attention played a crucial role only when the exogenous cue failed to provide sufficient guidance. Due to the invalid exogenous cue, attention was directed to a distractor immediately after the search array appeared, which required an attentional switch from the distractor to the target in order to complete the search task. Such a switch process might rely on top-down control and require cognitive resources, making it vulnerable to interference from WM-guided attention. However, once the exogenous cue became valid, there was an extremely strong bottom-up attention that emerged, which directed attention toward the location of the search target. This effect was so potent that it overshadowed the role of WM-guided attention, which would typically direct attention toward the location of the search distractor. These findings suggested that WM-guided attention and exogenous attention were not in parallel with each other; on the contrary, they competed with each other, which supported the fact that WM-guided attention and exogenous attention shared some common mechanisms.

The results of Experiment 2 suggested that WM-guided attention operated in parallel with endogenous attention, though WM-guided attention shares similarities with endogenous attention as mentioned in the introduction. Why did WM-guided attention compete with more automated exogenous attention instead of competing with endogenous attention which is more susceptible to the influence of cognitive load? One possible influential effect could be the paradigm adopted in our study, which reflected the automatic guidance of attention by WM. In the search task of this paradigm, the information consistent with the memorized item always appeared on the distractor rather than the target. As a result, the orienting of attention by WM actually disrupted the search, making it impossible for the participants to actively direct their attention according to WM. Therefore, the WM-driven attentional bias effect observed in our study might only reflect the automatic aspect of WM-guided attention, which explains why it is modulated by the exogenous cue. However, if a specific search task context is created, such as adding a target match condition to the search task, where information consistent with the memorized item may appear on the search target, the participants would have a strategic incentive to actively use their memorized information to guide attention, ultimately improving their search performance [7,53,54]. In this case, the WM-driven attentional bias effect could reflect the voluntary aspect of WM-guided attention and might be modulated by the endogenous cue.

Furthermore, it is important to note that the current findings are not only influenced by the task context of the search task, as discussed above, but are also limited to the particular type of search task employed in this study. One study used an individual differences approach to examine whether different types of search tasks probed the same underlying processes of how WM contents guide attention and found that two very similar search tasks were measuring the unique cognitive construct [55]. Specifically, the WM-driven attentional bias effect observed in the binary-stimulus search task (which was used in this study and required searching for the tilted line target among vertical or horizontal line distractors, each embedded within a different colored shape) did not correlate with the unitary-stimulus search task (which required searching for a top or bottom gap target among right or left gap distractors), and subsequent exploratory regression analyses revealed that the WM-driven attentional bias effect observed in the former search task could be predicted by visual WM capacity, while the latter could be predicted by attentional control and flexibility. This suggested a fundamental difference in how each search task taps into WM biasing, even within the same individuals. Therefore, the nature of WM-guided attention operating in other search tasks (e.g., the unitary-stimulus search task) may differ and require further exploration.

Based on the suggestion of an anonymous reviewer, it is worthwhile to further discuss whether the results of the current study hold true in more complex situations, such as when the participants are required to complete a more complex WM task, specifically a 3-back task. Typically, in the WM-guided attention paradigm, a simple WM task, such as a single-color change detection task, is used. However, if we were to use a 3-back task, it is important to note that the participants would have to remember at least three colors. As holding multiple pieces of information in WM can potentially limit their ability to guide attention [27,28], this aspect should be carefully considered. Moreover, even if we were to use a more complex WM task requiring the participants to remember only a single color, we should emphasize that the increased complexity of the WM task may strengthen the WM representation, but it may not directly enhance the WM-driven attentional bias effect. In fact, the WM representation required by simple WM tasks is already sufficient to produce this effect, and higher WM representation required by more complex WM tasks may not further modulate it. Therefore, we speculate that using a more complex WM task instead of an easy WM task may not result in significantly different findings for this study.

In sum, the current study found that the WM-driven attentional bias effect was modulated by the exogenous cue but not by the endogenous cue. This indicated that WM-guided attention competed with exogenous attention but not with endogenous attention, suggesting that WM-guided attention shared some mechanisms with exogenous attention, albeit to a certain extent, while operating in parallel with endogenous attention.

## Data Availability

Data are available from the corresponding author on reasonable request.

## Supplementary Materials

The following supporting information can be downloaded at: https://www.mdpi.com/article/10.3390/bs13050426/s1, Table S1: The accuracy for each condition in Experiments 1 and 2 (mean (SD) in %). Supplementary between-experiment comparison results.
