# Supplementary material for: Working-Memory-Guided Attention Competes with Exogenous Attention but Not with Endogenous Attention
Source: Behav Sci (Basel). 2023 May 18;13(5):426. doi: 10.3390/bs13050426 (PMC10215090; doi:10.3390/bs13050426)
Supplement: Supplementary file 1 [file behavsci-13-00426-s001.zip › behavsci-2376443-supplementary.pdf]

Supplementary material

	Experiment	Condition			
		WM match-cue invalid	WM unmatch-cue invalid	WM match-cue valid	WM unmatch-cue valid
Search task	1	98.57 (1.76)	98.73 (1.79)	99.39 (2.09)	99.02 (2.31)
	2	98.28 (2.74)	97.43 (3.08)	98.90 (1.27)	98.82 (1.61)
WM task	1	98.24 (2.22)	97.63 (2.33)	98.16 (3.11)	98.53 (2.27)
	2	96.32 (3.52)	96.81 (2.91)	97.06 (2.79)	98.49 (1.69)

Supplementary Table 1. The accuracy for each condition in Experiments 1 and 2 [Mean (SD) in %].

Supplementary between-experiment comparison results

The between-experiment three-way repeated-measures ANONA yielded a significant main effect of cue type ($F(1, 66) = 11.719$, $p = .001$, $\eta_p^2 = 0.151$), where the search RT was faster in the exogenous cue condition than that in the endogenous cue condition (803 ms vs. 847 ms). There was a significant main effect of cue validity ($F(1, 66) = 94.324$, $p < .001$, $\eta_p^2 = 0.588$), with faster search RT in the valid condition than that in the invalid condition (766 ms vs. 917 ms). There was also a significant main effect of WM-Search Match condition ($F(1, 66) = 36.461$, $p < .001$, $\eta_p^2 = 0.356$), with a slower search RT in the match than that in the mismatch condition (843 ms vs. 808 ms), indicating a WM-driven attentional bias effect. No significant two-way interaction was observed between cue type and WM-Search Match condition ($F(1, 66) = 1.315$, $p = .256$, $\eta_p^2 =$

0.020, $BF_{01} = 2.615$), nor between cue validity and WM-Search Match condition ($F(1, 66) = 0.661$, $p = .419$, $\eta_p^2 = 0.010$, $BF_{01} = 3.003$). The two-way interaction between cue type and cue validity was a significant ($F(1, 66) = 15.404$, $p < .001$, $\eta_p^2 = 0.189$). Both the exogenous cue and the endogenous cue produced a cueing effect, with the search RT being slower in the invalid condition than that in the valid condition (exogenous: MD = 90 ms, 95% CI [75, 106], $t(67) = 11.846$, $p < .001$, Cohen's $d = 0.621$; endogenous: MD = 213 ms, 95% CI [170, 255], $t(67) = 10.003$, $p < .001$, Cohen's $d = 1.187$), and the magnitude of the cueing effect produced by the endogenous cue being larger than that of the exogenous cue. More specifically, in the invalid-match condition, the search RT was faster for the exogenous than endogenous cue (MD = 181 ms, 95% CI [107, 255], $t(66) = 4.883$, $p < .001$, Cohen's $d = 1.184$), which was also the case in invalid-mismatch condition (MD = 188 ms, 95% CI [112, 263], $t(66) = 4.967$, $p < .001$, Cohen's $d = 1.205$). In the valid-match condition, no significant difference in the search RT was observed between the exogenous and endogenous cue condition (MD = 77 ms; 95% CI [-10, 163], $t(66) = 1.767$, $p = .082$, Cohen's $d = 0.429$; $BF_{01} = 1.074$), which was also the case in the valid-mismatch condition (MD = 47ms, 95% CI [-33, 128], $t(66) = 1.172$, $p = .245$, Cohen's $d = 0.284$; $BF_{01} = 2.238$).
